# Supplementary material for: In Vitro Infection Dynamics of Wuxiang Virus in Different Cell Lines
Source: Viruses. 2022 Oct 28;14(11):2383. doi: 10.3390/v14112383 (PMC9699334; doi:10.3390/v14112383)
Supplement: Supplementary file 1 [file viruses-14-02383-s001.zip › viruses-1933770-supplementary.pdf]

Supplemental Table S1. Primers for amplifying the ORF of M and L fragments of SXYQ1872-1.

	**Target sequence**	**Primer**	**Sequence (5'-3')**
WUXV-M	M-1	M1_F_$_{1-23}$	GCATGCATGTTTGAAATAATAGTAGTACT
		M1_R_$_{1082-1106}$	TGGGAATTGAAGAATGGGTATCTGT
	M-2	M2_F_$_{126-150}$	GTTCCTTTGGATAGAGAGTTACTGG
		M2_R_$_{1231-1253}$	CCTCTGGGACACTTATATGCCAT
	M-3	M3_F_$_{1167-1192}$	GACATTCTGTGATAGTRCAAAGATGT
		M3_R_$_{2127-2151}$	GTGCACCTTATAATGTGAGCTAACT
	M-4	M4_F_$_{2078-2098}$	GATCAGTRGGAGGTGATGTGG
		M4_R_$_{3034-3056}$	TCAACACAGCTGAACACCTCTAT
	M-5	M5_F_$_{2897-2921}$	CAGGAATGACAAAYAACACTGTGAT
		M5_R_$_{3970-3993}$	ATAGCCWATTATGATGCCAGCTGT
	M-6	M6_F_$_{3867-3888}$	GACACARGCTGCAAAGTCAATA
		M6_R_$_{4283-4301}$	TGACACAAAGACCGGTGCATAA
WUXV-L	L-1	L1_F_$_{1-19}$	ATGAATTCCATAATAGAGC
		L1_R_$_{1029-1054}$	AGTCTCCTTGGATACTTAGATCTGAT
	L-2	L2_F_$_{142-163}$	GTGGATAGAGAGCACTTTGACG
		L2_R_$_{1283-1307}$	CTCTGATCTCTTGACTCTTTAACGT
	L-3	L3_F_$_{1217-1242}$	TTTAGAAGAGTCCACCTATATTGCC
		L3_R_$_{2191-2215}$	GATTAAACATGCCCATCCATGAGAT
	L-4	L4_F_$_{2099-2121}$	CAGAGTTGCAGGTCCTAATCATG
		L4_R_$_{3038-3061}$	GTATGATCATTGGGTGCCATCTAG
	L-5	L5_F_$_{2889-2910}$	AATGAGAATTCCTGAGAGCCAC
		L5_R_$_{3962-3984}$	AAATTCCTCCTCATCACTACGCA
	L-6	L6_F_$_{3900-3921}$	AATGCAGTGACCAGGACAGAT
		L6_R_$_{4886-1908}$	CTTTATAAGTTCTGCCCACATGC
	L-7	L7_F_$_{4795-4816}$	GGTCTAAGCAAACTGTCAGTCG
		L7_R_$_{5787-5808}$	AGTCTCAGTGCAGGTCTTCAC
	L-8	L8_F_$_{5562-5586}$	GAACATATTGTCATACACAGCAGGT
		L8_R_$_{6250-6273}$	TCAGATGAAACCTTCAGAGTCACT

Supplemental Table S2. Different amino acid sites of M segment

**position**	**11**	**13**	**18**	**37**	**42**	**44**	**95**	**107**	**118**	**150**	**162**
SXWX1813-2	I	E	M	D	N	L	K	K	T	T	N
SXYQ1872-1	L	G	T	G	D	S	R	E	I	A	S
**position**	**172**	**177**	**227**	**257**	**269**	**406**	**517**	**548**	**703**	**1003**	**1066**
SXWX1813-2	A	V	T	M	P	I	I	V	A	V	I
SXYQ1872-1	P	I	A	I	S	T	T	I	T	I	M
**position**	**1081**	**1101**	**1168**	**1169**	**1184**						
SXWX1813-2	R	V	I	I	M						
SXYQ1872-1	K	I	V	V	V						

Supplemental Table S3. Different amino acid sites of L segment

**position**	**101**	**193**	**290**	**576**	**769**	**1161**	**1414**	**1597**	**1549**	**1594**	**1878**
SXWX1813-2	R	I	V	T	V	G	V	A	I	I	S
SXYQ1872-1	K	V	I	M	I	D	I	V	V	T	T

Supplemental Table S4. Information about primers and probe sequences of qRT-PCR for WUXV detection

Primers and Probe	Sequence（5’~3’）	Position*
WUXV_S-F	TTGGCAACAAGAGCCTTCAC	1346-1365
WUXV_S-R	ACAAGCCAAAGAAGATGCAAGA	1394-1415
WUXV_S-Probe	Cy5-TCCTTGGCACCCTCTGGAGACATCCT-BHQ2	1367-1392

Note: *The primers and probe locations in the genome were based on the S gene sequence of SXYQ1872-1 WUXV strain (GenBank Access No.: MT786487.1).
